# Rapid and Safe Neutralization Assay for Circulating H5N1 Influenza Virus in Dairy Cows

**DOI:** 10.1111/irv.70048

**Published:** 2024-12-02

**Authors:** Kei Miyakawa, Makoto Ota, Kaori Sano, Fumitaka Momose, Takashi Okura, Noriko Kishida, Tomoko Arita, Yasushi Suzuki, Masayuki Shirakura, Hideki Asanuma, Shinji Watanabe, Akihide Ryo, Hideki Hasegawa

**Affiliations:** ^1^ Research Center for Influenza and Respiratory Viruses National Institute of Infectious Diseases Tokyo Japan; ^2^ AIDS Research Center National Institute of Infectious Diseases Tokyo Japan; ^3^ Department of Microbiology Yokohama City University School of Medicine Kanagawa Japan; ^4^ Center for Emergency Preparedness and Response National Institute of Infectious Diseases Tokyo Japan; ^5^ Department of Virology III National Institute of Infectious Diseases Tokyo Japan

**Keywords:** dairy cows, H5N1, hiVLP, neutralization assay

## Abstract

Rapid and safe neutralization assays are required for highly pathogenic avian influenza viruses, including a clade 2.3.4.4b H5N1 subtype recently found in cows. Here, we report a neutralization assay using luminescent virus‐like particles. This assay has lower biosafety requirements and provides a larger dynamic range than conventional methods. We applied this technique to evaluate the cross‐reactivity of neutralizing antibodies induced by clade 2.3.4.4b candidate vaccine viruses (CVVs) with the cow‐derived H5N1 virus. Our findings indicate that these CVVs share antigenic characteristics with the cow‐derived H5N1 virus, suggesting the potential efficacy of vaccines developed using these CVVs.

## 1 Introduction

Highly pathogenic avian influenza (HPAI) viruses pose a significant threat to poultry and human health.^1^, ^2^ To mitigate these threats, several countries have stockpiled vaccines in preparation for potential pandemics.^3^, ^4^ In March 2024, a HPAI virus (subtype H5N1, clade 2.3.4.4b) was detected in dairy cows in the United States,^5^ with reports of transmission to humans.^6^, ^7^ Cows infected with this virus exhibit decreased milk production over a period of about 2 weeks, with the mammary glands and udder becoming swollen or sunken, but are typically known to have a low fatality rate in cattle.^7^ This unexpected host range expansion of the H5N1 virus to dairy cattle highlights the need for rapid assessment of vaccine efficacy against newly emerging strains. Although the World Health Organization (WHO) has designated two candidate vaccine viruses (CVVs) for this clade, i.e, IDCDC‐RG78A and NIID‐002,^8^ it is crucial to rapidly determine the antigenic similarity between these CVVs and the HPAI viruses circulating in dairy cows to ensure effective vaccine readiness.

The virus neutralization test (VNT) is the gold standard for evaluating vaccine‐induced neutralizing antibodies. However, this test is time‐consuming and requires biosafety level 3 (BSL3) facilities to handle authentic HPAI viruses. To address these limitations, a pseudovirus‐based VNT (PVNT) has been developed.^9^, ^10^ However, a pseudovirus that mimics cow‐derived H5N1 viruses has not yet been reported.

**FIGURE 1:**
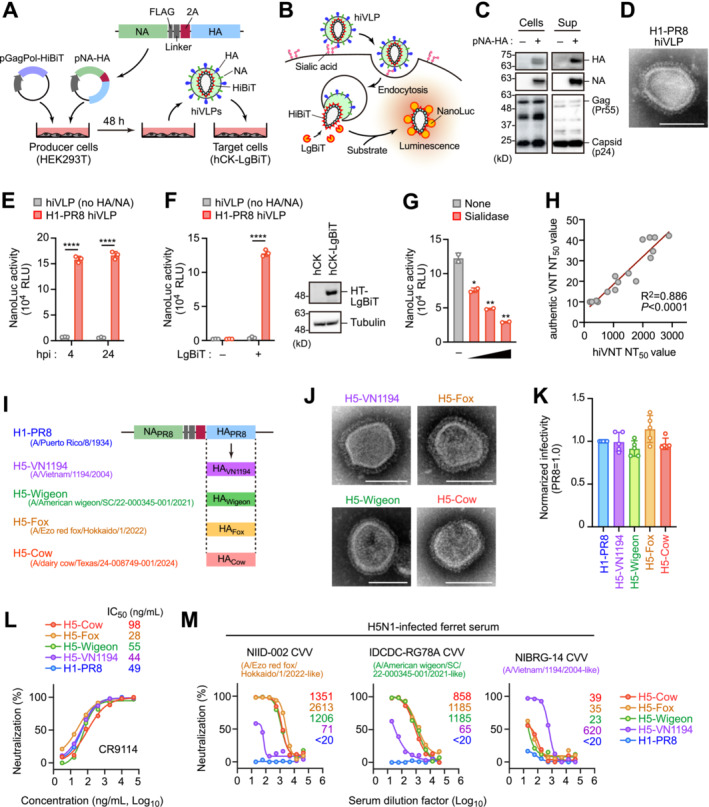
Development of rapid neutralizing test for influenza virus. (A, B) Generation of HiBiT‐tagged virus‐like particles (hiVLPs) mimicking influenza viruses. hiVLPs are produced by co‐expressing HIV‐1 GagPol genes fused with HiBiT and influenza virus HA and NA glycoprotein genes (pNA‐HA) in HEK293T cells (A). The virions emit light upon entry into LgBiT‐expressing target hCK cells (B). (C) Western blotting of virion‐producing cells and their culture supernatants (Sup), probed with anti‐HA, NA, and HIV‐1 Gag antibodies. (D) Electron microscopy image of the H1‐PR8 hiVLP. Scale bar, 100 μm. (E) Luminescence of hCK‐LgBiT cells after 4 and 24 h of indicated hiVLP infection (*n* = 3). (F) Luminescence in hiVLP‐infected hCK cells in the presence and absence of LgBiT (*n* = 3). Expression of LgBiT was confirmed by Western blotting and the representative data of two experiments are shown. (G) hCK‐LgBiT cells pretreated with sialidase (50, 500, and 5000 U/mL) were infected with hiVLPs (*n* = 2). Cell luminescence was measured 4 h after infection. (H) Correlation of 50% neutralizing titer (NT_50_) calculated by conventional virus neutralization test (VNT) and hiVLP‐based VNT (*n* = 16). (I) Construction of pNA‐HA plasmid series encoding HA sequence from H5‐VN1194 (A/Vietnam/1194/2004), H5‐Wigeon (A/American wigeon/South Carolina/22–000345‐001/2021), H5‐Fox (A/Ezo red fox/Hokkaido/1/2022), and H5‐Cow (A/dairy cow/Texas/24–008749‐001/2024). *Note:* NA was equivalent to H1‐PR8. (J) Electron microscopy image of the hiVLPs shown. Scale bar, 100 μm. (K) Normalized infectivity of indicated hiVLPs. Luminescence of hiVLP‐infected hCK‐LgBiT cells was normalized based on particle volume (HiBiT activity) (*n* = 4 or 5). (L) Neutralization curve and 50% inhibition concentration (IC_50_) values of the HA‐specific broadly neutralizing antibody, CR9114, against indicated hiVLPs (*n* = 2). Representative data are shown. (M) Neutralization curves and NT_50_ values for each ferret sera using the indicated hiVLPs (*n =* 2). Representative data are shown.

We previously developed a PVNT for SARS‐CoV‐2 using HiBiT‐tagged virus‐like particles (hiVLPs),^11^ and are currently applying this technology to influenza viruses. HiBiT, a small (11 amino acids) protein tag, forms a highly sensitive bioluminescent enzyme called NanoLuc luciferase when combined with its complementary partner LgBiT in target cells. This allows for the quantitative analysis of neutralizing antibodies by detecting luciferase activity. Our hiVLPs comprise the HIV‐1 GagPol core protein fused with HiBiT, forming self‐assembling, nonreplicating, and nonpathogenic particles (Figure 1A). Upon viral entry into susceptible hCK cells^12^ expressing LgBiT, it becomes reconstituted into a functional luciferase (Figure 1B). The resulting luminescence signal, detectable within 4–24 h postinfection, directly correlates with the number of viral particles entering cells. This approach allows for the rapid quantification of viral entry in BSL2 facilities.

To generate hiVLPs bearing surface glycoproteins of the influenza virus, HEK293 cells were co‐transfected with vectors encoding hemagglutinin (HA), neuraminidase (NA), and HiBiT‐fused GagPol (Figure 1A). First, we used the HA and NA glycoproteins from the well‐studied H1N1 strain PR8 (A/Puerto Rico/8/1934). Our experiments revealed that the 2A sequence‐linked co‐expression of HA and NA resulted in efficient and functional incorporation of both proteins into virions (Figure 1A). Subsequently, hiVLPs (designated as H1‐PR8) were harvested from HEK293T producer cells, and their expression in cells and virions was confirmed using western blotting (Figure 1C) and electron microscopy (Figures 1D and S1A). Upon the addition of virions to LgBiT‐expressing hCK cells,^12^ cell luminescence was observed within 4 h and remained stable for up to 24 h (Figure 1E). This luminescence was not observed in hCK cells lacking LgBiT expression or in sialidase‐treated cells (Figure 1F,G). Furthermore, treatment with a clathrin‐mediated endocytosis inhibitor (PitStop 2) attenuated luminescence without observable cytotoxicity (Figure 1B). These results indicate that H1‐PR8 virions can enter cells through mechanisms analogous to those of the authentic influenza viruses.

To evaluate the efficacy of hiVLPs as surrogates for authentic viruses in neutralization assays, we measured the 50% neutralization titer (NT_50_) in randomly selected human serum samples using both authentic viruses and hiVLPs. The results revealed a significant correlation (*R* ^2^ = 0.886, *p* < 0.0001) between the two methods (Figure 1H). Notably, the NT_50_ values calculated with authentic viruses ranged from 10 to 50, whereas those calculated with hiVLPs expanded to a range of 10 to 3000, suggesting that our hiVLP‐based PVNT provides a larger dynamic range when compared to the conventional VNT.

Subsequently, we adapted the hiVLP‐based PVNT to the cow‐derived HPAI virus by substituting the HA proteins in the H1‐PR8 hiVLP with those derived from cows and termed it as H5‐Cow hiVLP (Figure 1I). We also generated CVV‐derived hiVLPs (A/Vietnam/1194/2004 [H5‐VN1194] for clade 1, A/American wigeon/South Carolina/22‐000345‐001/2021 [H5‐Wigeon], and A/Ezo Red Fox/Hokkaido/1/2022 [H5‐Fox] for clade 2.3.4.4b), which are known as the original strains of NIBRG‐14, IDCDC‐RG78A, and NIID‐002 CVVs, respectively (Figure 1I). We confirmed the formation of virions carrying H5 HA using electron microscopy (Figure 1J), and these particles exhibited efficient infectivity (Figure 1K). The HA‐specific broadly neutralizing antibody, CR9114, which shows cross‐reactivity to influenza virus subtypes,^13^ significantly inhibited the cell entry of these virions (Figure 1L). Notably, ferret sera immunized with IDCDC‐RG78A and NIID‐002 CVVs significantly inhibited infection of H5‐Cow virions, as well as H5‐Wigeon and H5‐Fox virions (Figure 1M). Additionally, two ferret sera against H5N1 clade 2.3.4.4b recently isolated in Japan (A/chicken/Kagoshima/21A6T/2021 and A/chicken/Kagawa/22A9T/2022) also displayed good protection against H5‐Cow virions (Figure S2). Conversely, antisera against the NIBRG‐14 CVV exhibited minimal or no protection against the H5‐Cow virions (Figure 1M). These results suggest that currently circulating H5N1 virus in dairy cows shares antigenic characteristics with IDCDC‐RG78A, NIID‐002, and recently isolated H5N1 clade 2.3.4.4b viruses.

This study had several limitations. First, the hiVLPs used in this study contained N1 from the PR8 strain. The N1 from PR8 may differ from the N1 of the current H5N1 strain in terms of antigenicity, potentially impacting result interpretation. In future studies, it will be necessary to use a relevant N1 and to investigate the effects of NA in further detail. Nonetheless, given that most broadly neutralizing antibodies bind to the highly conserved stem region of HA among influenza virus subtypes,^14^, ^15^ most neutralizing antibodies should be detectable using our hiVLP‐based PVNT. Second, the structure of pseudoviruses differs from that of authentic influenza viruses. Pseudoviruses possess surface proteins (HA and NA) of influenza viruses, but their other structures are significantly different. Therefore, it is unclear whether all of the signal is due to neutralizing antibody response or antibody binding to epitopes that are exposed on a pseudovirus format that would not be accessible on an authentic influenza virus particle. Further research is needed to fully understand the relationship between hiVLP‐based PVNT and authentic virus VNT readouts.

In conclusion, the vaccines developed using H5N1 clade 2.3.4.4b CVVs exhibit potential efficacy against cow‐derived HPAI viruses. Moreover, the lower biosafety requirements of hiVLP‐based PVNT make it accessible to a broader range of research facilities, potentially accelerating vaccine development and epidemiological studies.

## Author Contributions

**Kei Miyakawa:** writing – original draft, funding acquisition, investigation, conceptualization, methodology, project administration. **Makoto Ota:** investigation. **Kaori Sano:** investigation, resources. **Fumitaka Momose:** investigation, resources. **Takashi Okura:** resources. **Noriko Kishida:** resources. **Tomoko Arita:** resources. **Yasushi Suzuki:** resources. **Masayuki Shirakura:** resources. **Hideki Asanuma:** resources. **Shinji Watanabe:** resources. **Akihide Ryo:** supervision. **Hideki Hasegawa:** supervision, funding acquisition.

## Conflicts of Interest

The authors declare no conflicts of interest.

## Supporting information


**Figure S1.** Characteristics of the hiVLPs.
**Figure S2.** Neutralization activity of recently isolated H5N1‐infected ferret serum.
**Data S1.** Supplementary Information.

## Data Availability

The data that support the findings of this study are available from the corresponding author upon reasonable request.
